# “It’s Got to Be on This Page”: Age and Cognitive Style in a Study of Online Health Information Seeking

**DOI:** 10.2196/jmir.3352

**Published:** 2015-03-24

**Authors:** Emily M Agree, Abby C King, Cynthia M Castro, Adrienne Wiley, Dina LG Borzekowski

**Affiliations:** ^1^Johns Hopkins UniversityDepartments of Sociology and Population, Family, and Reproductive HealthBaltimore, MDUnited States; ^2^Stanford Prevention Research CenterSchool of MedicineStanford UniversityPalo Alto, CAUnited States; ^3^Kauffman AssociatesWashington, DCUnited States; ^4^Dept of Behavioral and Community HealthSchool of Public HealthUniversity of MarylandCollege Park, MDUnited States

**Keywords:** eHealth, Internet, health literacy, age groups, field dependence-independence

## Abstract

**Background:**

The extensive availability of online health information offers the public opportunities to become independently informed about their care, but what affects the successful retrieval and understanding of accurate and detailed information? We have limited knowledge about the ways individuals use the Internet and the personal characteristics that affect online health literacy.

**Objective:**

This study examined the extent to which age and cognitive style predicted success in searching for online health information, controlling for differences in education, daily Internet use, and general health literacy.

**Methods:**

The Online Health Study (OHS) was conducted at Johns Hopkins School of Public Health and Stanford University School of Medicine from April 2009 to June 2010. The OHS was designed to explore the factors associated with success in obtaining health information across different age groups. A total of 346 men and women aged 35 years and older of diverse racial and ethnic backgrounds participated in the study. Participants were evaluated for success in searching online for answers to health-related tasks/questions on nutrition, cancer, alternative medicine, vaccinations, medical equipment, and genetic testing.

**Results:**

Cognitive style, in terms of context sensitivity, was associated with less success in obtaining online health information, with tasks involving visual judgment most affected. In addition, better health literacy was positively associated with overall success in online health seeking, specifically for tasks requiring prior health knowledge. The oldest searchers were disadvantaged even after controlling for education, Internet use, general health literacy, and cognitive style, especially when spatial tasks such as mapping were involved.

**Conclusions:**

The increasing availability of online health information provides opportunities to improve patient education and knowledge, but effective use of these resources depends on online health literacy. Greater support for those who are in the oldest cohorts and for design of interfaces that support users with different cognitive styles may be required in an age of shared medical decision making.

## Introduction

### Background

Making informed decisions about health and health care is a key part of enhanced patient care in the twenty-first century. Shared decision making between patients and providers is increasingly the preferred model for health care delivery [1,2]. Improved patient involvement in medical decision making allows more informed decisions overall, as well as better patient perception of medical risks, greater patient engagement, and increased cooperation and compliance among patients [3,4]. For more involvement, patients not only need access to scholarly and popular online health information, but they must also be able to understand the retrieved information [5]. Critical to shared decision making is patients’ successful online health seeking where they find “transparent and credible information about the relative value and risk of various medical diagnostic and therapeutic interventions” [6].

Since the Internet’s introduction, a common activity has been seeking health information online. At the time of the first Health Information National Trends Survey (HINTS) in 2005, 58% of Internet users had used the Internet to search for health information for themselves [7]. By the fourth wave of HINTS in 2012, this had risen to 77.6% [8]. Websites and online forums offer opportunities to obtain and evaluate information about almost every imaginable health condition, treatment, and/or test. Yet, our knowledge of how individuals retrieve and understand online health information is still limited; we also know little about how individuals’ use of online tools affects treatment choices and health outcomes [9]. Research shows that more intensive Internet users prefer greater involvement in medical decisions, but even among these intensive users individual ability to obtain and process online information varies widely [10]. For example, older individuals experiencing cognitive changes and sensory limitations tend to have difficulties interacting with computers and accessing online resources.

### Online Health Literacy and Aging

The ability of individuals to participate in informed decisions about their health care depends on the degree to which they have the capacity to obtain, process, and understand health information (health literacy) [11,12]. Online health literacy (sometimes called *eHealth literacy*) is simply the ability of individuals to obtain, process, and understand health information from the Internet.

Older adults are a particularly vulnerable group, characterized by low health literacy and poor health outcomes [13]. Gazmararian and colleagues [14] reported one-third of Medicare managed-care enrollees had difficulty reading written information from health care providers, with the lowest health literacy among those aged 85 years and older. Low health literacy among older adults is compounded by the fact that older adults experience more chronic diseases, take more medications, and visit health care providers more often than younger adults. Often, health information presented to older adults is complex, reflecting multiple health concerns and conditions. As we age, our search for health information can be more demanding; as a result, the Internet’s role as a primary repository becomes critically important [15].

**Figure 1:**
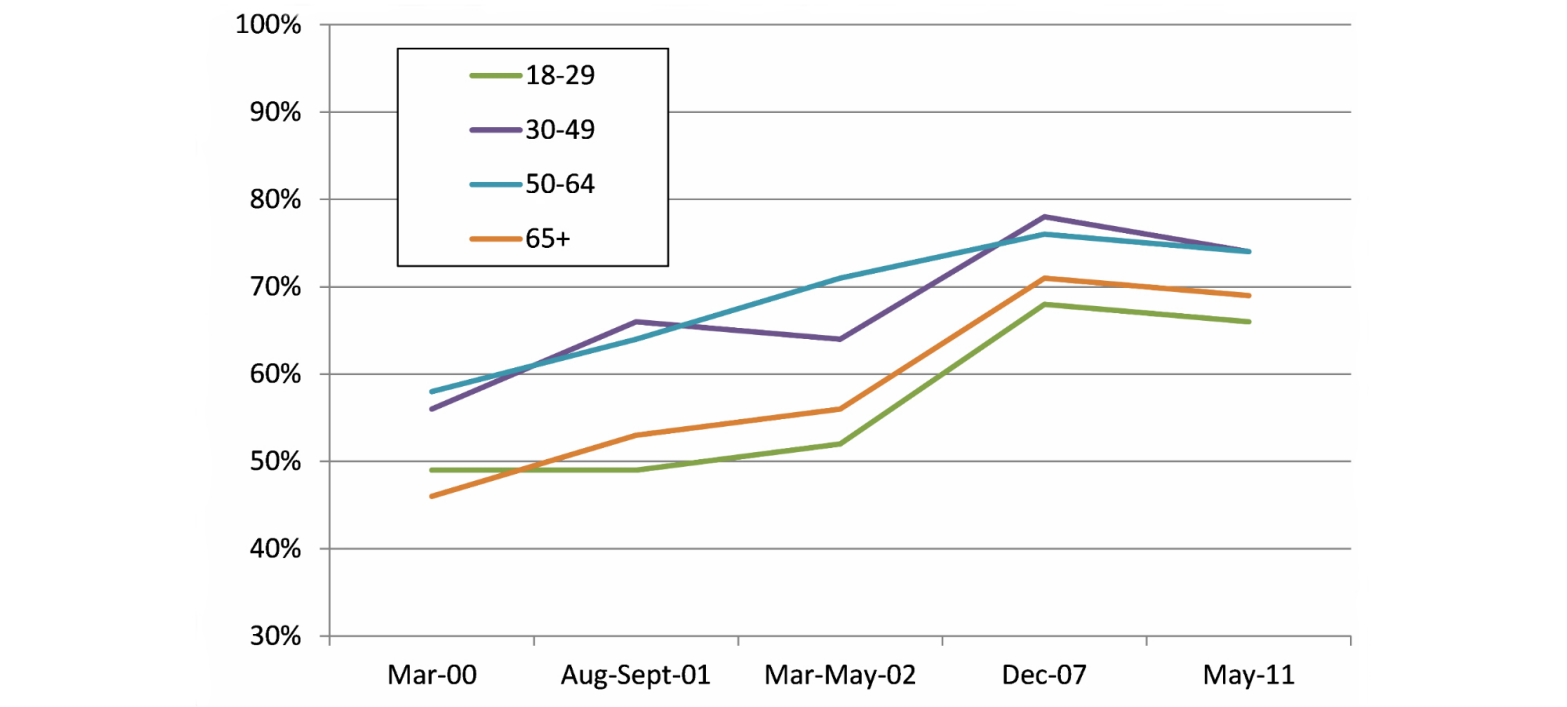
Percentage of Internet users who looked for health information online. Tabulations are drawn from the Pew Internet and American Life Project spreadsheet “Usage Over Time” [18].

Age differences in computer use, skills, and Internet use are well established. Older cohorts are far less likely than younger ones to use computers regularly, more rarely rely on the Internet for information, and report less ease in locating information on the “net” [16]. Despite these differences, the closing of the age-based digital divide is greatly anticipated as new cohorts, more familiar with computers, enter old age. The proportion of adults using the Internet has steadily risen among adults of all ages. For those aged 65 and older, the rate has risen from 13% to almost 60% in just 10 years [17]. The proportion of Internet users who look for health information online also increased over the same period (Figure 1). Those at the oldest and youngest ages are the least likely to seek health information online—the oldest perhaps because they are less trusting of online resources and the youngest because they are less in need of health information, in general.

Basic computer skills—as well as the ability to discriminate among online resources and understand and use information—are paramount for user success obtaining online health information. Age-related declines in sensory abilities and cognition affect visual acuity, especially the ability to discriminate important information in a graphically challenging visual field. Such declines also serve as key factors influencing the usefulness of online resources for older persons [18].

### Cognitive Style

Cognitive style reflects the different ways individuals solve problems; people vary in how they acquire and process information [19]. A central distinction in the measure of cognitive style is between individuals who are context independent or context sensitive. Those who are context independent are better able to abstract a visual element from its background than those who are context sensitive. Context-independent people often have a more analytic approach to learning; they tend to outperform context-sensitive people in structured learning environments [20]. Persons who may be described as context sensitive observe less differentiation between an object and its context. They tend to be more global in their approach to information and more responsive to visual cues, such as color coding of information [21,22]. Studies show that both context-independent and context-sensitive individuals can perform equally well, but approach learning and use different tools [20,23].

How cognitive style affects success in navigating the Internet deserves increased examination, given the increasing dependency of decision making on online information. Not all websites are the same. Many use increasingly complex interfaces and rely on multimedia content to convey information [24]. Search engine interfaces and the presentation of search results also have received little attention in terms of design and organization [25]. Although accessibility guidelines address sensory and physical deficits, website and search engines are not designed to accommodate differences in learning styles. Some evidence suggests that context sensitivity may increase with age, as cognitive changes affect visual-spatial learning [26]. A better understanding of age-related changes in the perception of screen content, which can affect success in locating and understanding online health information, is needed.

In this study, we examined the extent to which cognitive style (as dichotomized by context independence vs sensitivity) matters for success with online health seeking, controlling for differences in age, education, health literacy, and Internet experience.

## Methods

### Study Design

The Online Health Study (OHS) was developed to explore age differences in the strategies used by adults to successfully navigate the Internet for health information and to understand how older adults’ online health literacy compares with that of younger adults. The OHS was designed to examine demographic, cognitive, and environmental factors associated with success obtaining online health information.

The study was conducted at the Johns Hopkins School of Public Health and Stanford University School of Medicine. From April 2009 to June 2010, 346 men and women aged 35 years and older of diverse racial-ethnic backgrounds participated. Participants were recruited from the community and screened for eligibility using a Web-based interface constructed by the research team. Such screening allowed appropriate representation in the sample from different demographic groups and ensured that potential participants had suitable levels of Internet skill to work through the study protocols. Participants who completed all study procedures received a US $35 gift card.

After completing consent procedures, participants provided information on their socioeconomic and demographic backgrounds, health status, and experience with computers and other media. Participants also completed a Rapid Estimate of Adult Literacy in Medicine (REALM) to provide a general measure of health literacy and the Witkin Group Embedded Figures Test (GEFT) to assess context sensitivity or independence.

**Figure 2:**
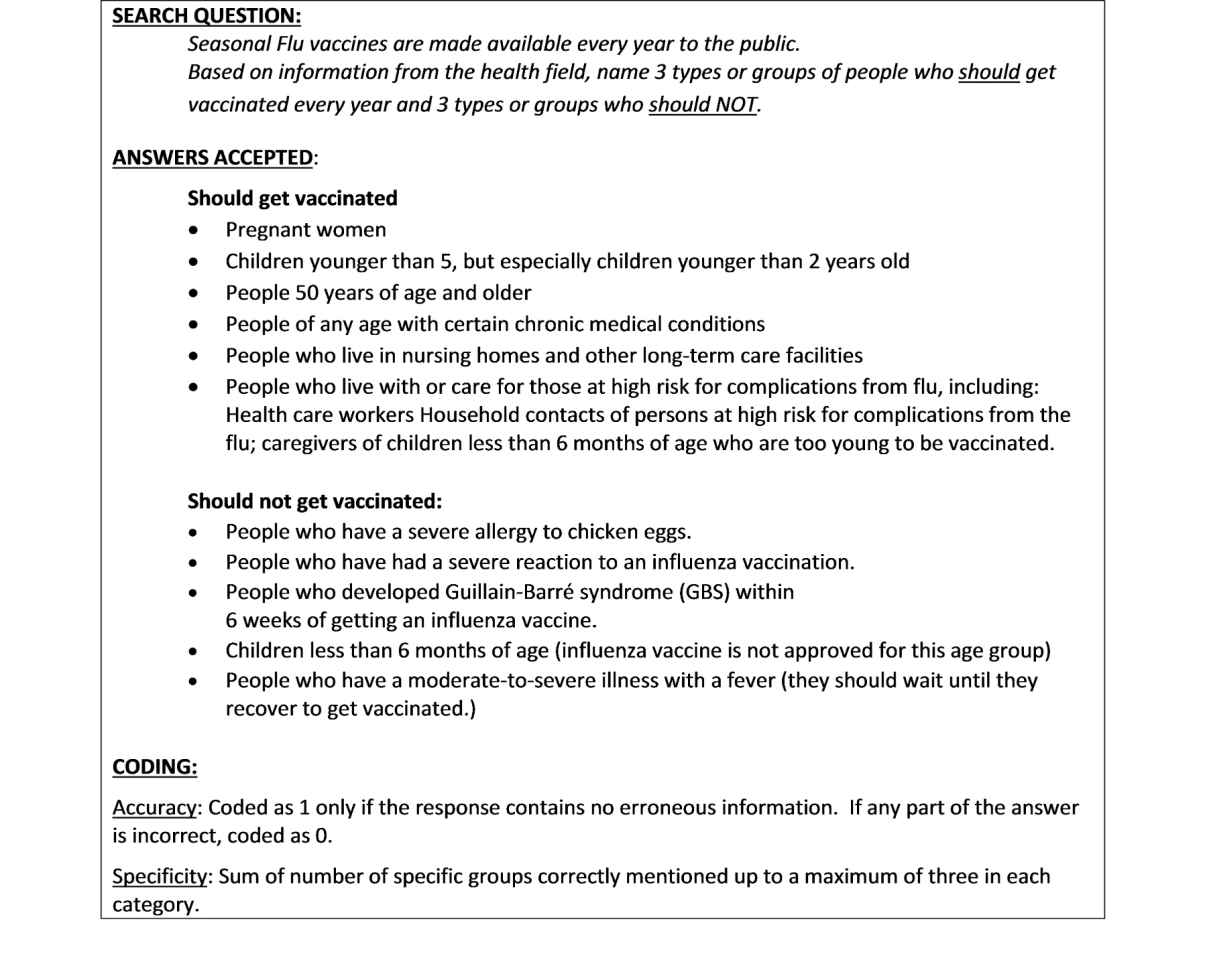
Example of an online health task and coding scheme for accuracy and specificity.

Participants did a practice search task to familiarize themselves with the protocol and warm-up to using the research computers. Then, participants answered 6 health-related questions by performing online searches on the Hopkins or Stanford project computers. Search time was limited to 15 minutes per task and sessions averaged 60-90 minutes overall. After each online search task, participants reported their answer, which was transcribed and later coded by 2 assistants for response accuracy and specificity. Online search tasks reflected typical and realistic tasks. Health topics covered in the search tasks included diet/nutrition guidelines, skin cancer, alternative medicine, vaccine recommendations, assistive health technology, and over-the-counter genetic testing. In addition to varying on subject matter, each of the tasks required different levels of health literacy and computing skills, including reading texts, reviewing charts, locating health resources on maps, performing simple computations, and evaluating diverse health opinions. For example, the nutrition question asked participants to name 2 heart-healthy foods. This required reading recommendations and lists. The question for assistive technology involved online mapping skills, as participants were asked to locate a store near a specific address where grab bars could be purchased. Figure 2 offers an example of one of the OHS search tasks and the coding guidelines used to score accuracy and success.

### Analytic Approach

To examine the relative importance of age, education, health literacy, Internet use, and cognitive style for accuracy and success in online health searches, we analyzed a sample of 323 participants with complete data (93.1% of the 347 participants enrolled in the study) from both sites (128 from Johns Hopkins and 195 from Stanford).

Outcome measures included both overall accuracy (the number of accurate answers on each of the 6 search tasks) with a potential range of 0-6, and success—a scale that combined both accuracy (coded 0-1 for each item) and specificity (coded 0-2 for each item). The potential range for the success scale was 0-18. Models also were estimated for both outcomes with individual search tasks (range of 0-1 for accuracy and 0-2 for success). STATA 11 (StataCorp LP, College Station, TX, USA) was used to estimate generalized linear models (GLM), logistic regression, and ordinal logistic regression models as appropriate to the specific outcome.

Key predictors included an assessment of health literacy using the REALM [27]. The REALM is a vocabulary-based health literacy measure in which each participant reads aloud a list of 66 medical terms; scores are based on correctness of pronunciation. To accommodate the diversity of our study sample, each answer was coded by 2 different assistants as either “0” (incorrect), “1” (incorrect because of a non-English accent), or “2” (correct). The potential range of our coding of the REALM was 0-132.

Cognitive style was measured using the GEFT [19,28] The GEFT involves identification of a visual figure, such as a triangle, within a more complex line drawing. Correct answers are summed in a total score. Those who scored 12 or higher were classified as context independent (treated as the reference group in models), and those scoring 11 or lower were considered to be context sensitive. The GEFT has been validated across settings and age groups; it is also a nonverbal test, which reduces confounding effects of educational attainment. The GEFT complements the use of the REALM for health literacy, which relies on verbal abilities and vocabulary knowledge for assessment.

## Results

Participants’ ages ranged from 35-90 years, with a stratified design that ensured representation across broad age and sex groups (see Table 1). The sample had a mean age of 55.5 (SD 1.6) years, with approximately 25% (80/323, 24.8%) of the participants aged 60-69 years and 16.4% (53/323) aged 70 or older. More than half (193/323, 59.8%) were female. The sample was not designed to be representative of the entire population, but more closely resembles the general population of Internet users. Most participants were highly educated (65%, 208/323 with college or higher education), reported daily Internet use (88.3%, 285/323), and had good health literacy (REALM mean 128.5, SD 3.6). The mean score on the GEFT was 10.9 (SD 3.0), with 58.8% (190/323) of the sample classified as context sensitive.

**Table 1:** Participants in the Online Health Study (N=323).

Variable		Participants
**Age, n (%)**		
	35-59 years	190 (58.8)
	60-69 years	80 (24.8)
	≥70 years	53 (16.4)
**Sex, n (%)**		
	Male	130 (40.2)
	Female	193 (59.8)
**Education, n (%)**		
	High school or less	113 (35.2)
	College or more	208 (64.8)
**Daily Internet use, n (%)**		285 (88.3)
**Health literacy, mean (SD)**		
	REALM score	128.5 (3.6)
**Cognitive style, n (%)**		
	Context sensitive	190 (58.8)
	Context independent	133 (41.2)

On average, participants answered 4.1 of 6 search tasks with no errors and 29 of 323 participants (9.0%) gave accurate responses to all 6 questions. Performance on individual tasks varied substantially, with the highest percentage giving error-free answers for the question about heart-healthy foods (95.4%, 308/323) and the lowest on the flu question (32.5%, 105/323) (see Table 2). For success in online health seeking, which combined accuracy with the specificity of the responses, participants averaged a score of 8.2 out of 12. Our success measure showed similar variation across question types, with the most success on naming 2 heart-healthy foods and the worst for the question on whether herbs can help with memory loss. Although no question was specifically more difficult than another, they did test different types of health literacy and skills so that variation can be attributed in part to different types of Internet search skills.

**Table 2:** Scores for accuracy and success for the online health task (N=323).

Topic	Search task/question	Accuracy, %	Success, mean (SD)
Nutrition	Name 2 heart-healthy foods	95.4	1.8 (0.5)
Cancer	How do you identify skin cancer?	81.7	1.6 (0.6)
Alternative medicine	Can herbs help memory?	59.1	0.7(0.6)
Vaccinations	Who should get flu shots?	32.5	1.2 (0.6)
Assistive technology	Where can you find a store selling grab bars?	75.9	1.6 (0.8)
Genetic testing	Should genetic tests be sold over the counter?	70.0	1.3 (0.8)
Overall (all 6 items)		9.0	8.2 (1.8)

Table 3 shows regression models for success in online health seeking. In the first model, those in the oldest age group had significantly lower success scores relative to younger participants. There were no significant sex differences, although having a college degree and daily Internet use both associated positively with more successful health information searches. In Model 2, we added the REALM measure of health literacy, also significantly associated with better success in searching. In the final model, we added the indicator for cognitive style, which demonstrated a strong negative association with success. When controlling for education, health literacy, current Internet use, and context sensitivity throughout the models, the oldest individuals exhibited a significant disadvantage in the successful attainment of online health information.

**Table 3:** Coefficients for generalized linear models predicting overall search success.

Variables		Success, coefficient (95% CI)		
		Model 1	Model 2	Model 3
**Age**				
	35-59 years	Reference	Reference	Reference
	60-69 years	–0.36 (–0.84, 0.11)	–0.37 (–0.84, 0.10)	–0.32 (–0.79, 0.15)
	≥70 years	–0.71 (–1.28,–0.14)	–0.86 (–1.43, –0.29)	–0.78 (–1.34, –0.21)
**Sex**				
	Male	Reference	Reference	Reference
	Female	0.22 (–0.19, 0.62)	0.17 (–0.23, 0.57)	0.23 (–0.17,0.63)
**Education**				
	High school or less	Reference	Reference	Reference
	Some college or more	0.51 (0.07, 0.94)	0.44 (0.00,0.87)	0.36 (–0.07, 0.80)
**Daily Internet use**		0.94 (0.35, 1.54)	0.80 (0.21, 1.40)	0.80 (0.02, 0.13)
**Health literacy**				
	REALM score		0.09 (0.04, 0.15)	0.08 (0.22,1.39)
**Cognitive style**				
	Context independent			Reference
	Context sensitive			–0.58 (–0.99, –0.18)
Constant		7.12 (6.47, 7.77)	–4.29 (–11.31, 2.72)	–2.36 (–9.45, 4.72)

Table 4 displays ordered logistic regression models for success for each specific search task. Outcomes varied with respect to sex, daily Internet use, health literacy, and cognitive style when successfully seeking online health information. Those in the oldest age group showed a negative association with overall success for all the tasks, although the effects for age were significant only for a specific task involving mapping skills (wherein participants needed to locate a store selling grab bars). Being female was only significantly associated with the ability to report visual indicators of cancerous skin growths: women were more than twice as likely to be more successful than men, controlling for other variables. Having a higher education was positively associated with success across several—although not all—tasks. It was not associated with success for questions about heart-healthy food or public guides for identifying cancerous skin growths. Surprisingly, during the search task that asked participants to investigate the ability of herbal supplements to reduce memory loss, those with more education were less likely to be successful than those with less education.

**Table 4:** Odds ratios for ordered logistic regression models predicting success with specific online health-seeking tasks.

Variables		Online health-seeking task, OR (90% CI)					
		Nutrition	Cancer	Alternative medicine	Vaccinations	Mapping	Genetic testing
**Age**							
	35-59 years	Reference	Reference	Reference	Reference	Reference	Reference
	60-69 years	0.80 (0.47, 1.36)	0.92 (0.57, 1.50)	0.71 (0.46, 1.11)	1.21 (0.76, 1.92)	0.59 (0.34, 1.00)	0.85 (0.55, 1.32)
	≥70	0.59 (0.31, 1.10)	0.76 (0.42, 1.38)	0.60 (0.35, 1.03)	0.78 (0.44, 1.40)	0.44 (0.24, 0.83)	0.71 (0.42, 1.22)
**Female**		0.80 (0.50, 1.29)	2.33 (1.54, 3.54)	0.88 (0.60, 1.29)	1.08 (0.73, 1.61)	0.92 (0.58, 1.45)	1.35 (0.93, 1.96)
**Education**							
	High school or less	Reference	Reference	Reference	Reference	Reference	Reference
	College or more	0.98 (0.59, 1.62)	1.09 (0.69, 1.73)	0.34 (0.22, 0.53)	1.67 (1.08, 2.59)	2.00 (1.23, 3.27)	2.08 (1.40, 3.10)
**Daily Internet use**		1.77 (0.96, 3.25)	2.27 (1.28, 4.04)	3.73 (2.06, 6.75)	1.32 (0.72, 2.43)	0.84 (0.43, 1.62)	1.10 (0.64, 1.90)
**REALM**		1.02 (0.96, 1.09)	1.04 (0.99, 1.11)	0.95 (0.90, 1.01)	1.07 (1.01, 1.13)	1.04 (0.98, 1.11)	1.11 (1.06, 1.17)
**Cognitive style**							
	Context independent	Reference	Reference	Reference	Reference	Reference	Reference
	Context sensitive	0.54 (0.33, 0.88)	0.57 (0.37, 0.88)	0.73 (0.50, 1.08)	0.87 (0.58, 1.29)	0.59 (0.37, 0.96)	1.00 (0.68, 1.46)

Health literacy and cognitive style were significant for different outcomes. A higher REALM score was positively associated with success on the search task about the flu vaccine and over-the-counter genetic tests. Context sensitivity was negatively associated with naming heart-healthy foods, identifying cancerous skin growths, and locating a store that sells grab bars. Context-sensitive participants were roughly half as likely to be successful compared to context-independent individuals

## Discussion

The increasing availability of online health resources would suggest improved patient education and knowledge; however, this outcome requires successful online health literacy. Patients need to retrieve and comprehend online health information in order for it to positively impact decision making and health care. Results from this study suggest that the oldest health seekers may be at a disadvantage compared to younger cohorts, even after controlling for technology use, education, health literacy, and cognitive style, especially when spatial tasks such as mapping are involved.

Cognitive style was hypothesized to be particularly important for success in online health seeking. Our results reveal that context sensitivity was associated with less success in obtaining online health information, with specific tasks involving visual judgment and mapping most affected. These results are consistent with the idea that individuals who are context sensitive will indeed be most greatly affected by tasks that involve spatial abilities (such as using mapping tools to find a store or understanding the visual illustrations of skin cancer). They also seem to perform worse in the relatively straightforward identification of heart-healthy foods, in part because they tend to be less specific and thus more vague about the types of food items that are recommended.

In addition, better health literacy seems important both for overall success in searching and specifically for questions requiring prior health knowledge, such as flu vaccine recommendations or the sale of over-the-counter genetic tests. Interestingly, those with greater education were less likely to answer the question about alternative medicine correctly. This is consistent with other research that has shown that complementary and alternative medicine has been taken up at greater rates by those with higher education levels who are more skeptical of the allopathic medical profession [29].

Study limitations should be acknowledged. Although the GEFT has been used extensively in studies of hypermedia learning especially to examine differences in website design and other graphical interfaces [30], it also can be affected by participants’ visual acuity and other abilities. As with previous studies, we employ a dichotomous indicator of context sensitivity, but it may be more appropriate to examine cognitive style as a continuum [31]. The extent to which Internet searchers have prior knowledge about a topic can also affect their approaches to navigating the Internet [32]. Those with more expertise tend to seek out specific information, whereas novices benefit from a broader overview that orients them to the subject. Finally, this research evaluated participants’ online health seeking during a single monitored session. There is evidence that patients develop avenues for online health information over time, employing iterative processes that involve multiple sessions over longer time periods [33]. Some OHS study group participants may have been more successful in their online health seeking if given more time to search online or assisted by a family member or health provider.

The present study raises issues concerning online health communication, suggesting that vulnerable populations may need targeted assistance if this is to be a primary source of information in decision making. Older cohorts as well as those with different cognitive styles had difficulties finding accurate, detailed information. When posed very specific questions, some people can easily find information; others are lost, even when the information seems readily apparent on a particular webpage.

It is difficult to control the online health information to which people will be exposed. Websites change continuously and search results can yield different outcomes over a course of a single day. Health educators and providers can potentially serve as a resource, providing guidance to reliable and credible websites that maintain appropriate standards of content and usability [34]. They could also help interpret search findings, especially when patients obtain inaccurate, vague answers to the health questions. However, this recommendation requires that health educators and providers have sufficient time and skills to serve these functions, which may not be the case. Other community resources (such as librarians or adult educators) could possibly help deliver this support to those with low online health literacy.

Adaptations to health information websites could reduce distracting content and highlight key sections, as this has been shown to help persons with context sensitivity [35]. Websites about skin cancer could supplement visual information with verbal descriptions in order not to lose patients whose cognitive style is more context sensitive. In addition, educational research has shown that context-sensitive individuals benefit from additional levels of structure and guidance in information materials [36]. Thus, indexes (or more recent tools such as drop-down menus) might be useful additions to websites that would help these users [30].

However, the rising dominance of Google search engines as the primary interface for searching means that indexes and other website design tools may be receding in importance [37]. Thus, the promotion of standards for health information website design, similar to the accessibility standards aimed to help Internet users with disabilities, should be only one aspect of design taken into account. Rather than focusing on individual websites, information technologists could design applications such as browser add-ons or mobile apps that improve online interfaces and thus reduce some of the cognitive demands involved in searching. In light of the growing importance of and reliance on online health information-seeking across all age groups, such improvements may prove necessary and critical for improved public health.

## Abbreviations

GEFT: Group Embedded Figures Test
GLM: generalized linear model
HINTS: Health Information National Trends Survey
OHS: Online Health Study
REALM: Rapid Estimate of Adult Literacy in Medicine
